# Qualitative and quantitative analysis of the proautophagic activity of Citrus flavonoids from Bergamot Polyphenol Fraction

**DOI:** 10.1016/j.dib.2018.05.139

**Published:** 2018-05-31

**Authors:** Elzbieta Janda, Raffaele Salerno, Concetta Martino, Antonella Lascala, Daniele La Russa, Manuela Oliverio

**Affiliations:** a Department of Health Sciences, Magna Graecia University, Campus Germaneto, Catanzaro, Italy; b Interregional Research Center for Food Safety and Health, Catanzaro, Italy; c Department of Ecology, University of Calabria, Rende, Cosenza, Italy

## Abstract

Bergamot Polyphenol Fraction (BPF®) is a natural mixture of *Citrus* flavonoids extracted from processed bergamot fruits. It has been shown to counteract cardiovascular risk factors and to prevent liver steatosis in rats and patients. Hepatic effects of BPF correlate with its ability to stimulate liver autophagy. Six aglyconic flavonoids have been identified in the proautophagic fraction of the hydrolysis product of BPF (A-BPF): naringenin, hesperetin, eridictyol, diosmetin, apigenin and luteolin. We report here the output parameters of high resolution mass spectrometry analysis of these flavonoids and chemical structures of their parent compounds. The second set of data shows the proautophagic activity of BPF flavonoids in a hepatic cell line HepG2 analyzed by a flow cytometry approach. The method is based on the red to green fluorescence intensity ratio analysis of DsRed -LC3- GFP, which is stably expressed in HepG2 cells. Proportional analysis of ATG indexes allowed us to address a relative contribution of individual compounds to the proautophagic activity of the A-BPF mixture and evaluate if the effect was additive. Qualitative analysis of ATG indexes compared the effects of flavonoids at equal concentrations in the presence and absence of palmitic acid and chloroquine. The Excel files reporting the analysis of flow cytometry data are available in the public repository.

**Specifications Table**

**Table:** 

Subject area	Pharmacology and cell biology
More specific subject area	Flavonoid pharmacology and autophagy
Type of data	Tables, text file, graphs, dot-plots
How data was acquired	liquid chromatography- high resolution mass spectrometry (LC-HRMS) Q-ExactiveTM (Thermo Scientific) and flow cytometry (FACS Canto II, BD Biosciences)
Data format	Raw and analyzed
Experimental factors	Flavonoid aglycons and their mixtures, treatments for 6 h, chloroquine 2 h, palmitic acid added for 22 h and then withdrawn
Experimental features	GR-LC3-HepG2 cells (HepG2 cells expressing DsRed -LC3- GFP)
Data source location	Campus Germaneto, Catanzaro, Italy
Related research article	Lascala et al. *Analysis of proautophagic activities of Citrus flavonoids in liver cells reveals the superiority of a natural polyphenol mixture over pure flavones*[1]. https://doi.org/10.1016/j.jnutbio.2018.04.005

**Value of the Data**

• We provide output LC-HRMS parameters for naringenin, hesperetin, eriodictyol, diosmetin, apigenin and luteolin and the list of parent flavonoid glycosides found in BPF.
• A fast flow cytometry method to analyze autophagy is illustrated in a detailed manner and supported by row data, so it can be easily reproduced by other researchers.
• We describe here a “proportional” approach to the analysis of the proautophagic activities in a mix of compounds that can be applied to other mixtures.
• The autophagy index (ATG index) data for six typical flavonoid aglycones reported here, can be used as a reference for other cell lines and compounds.

## 1 Data

**Fig. 1:**
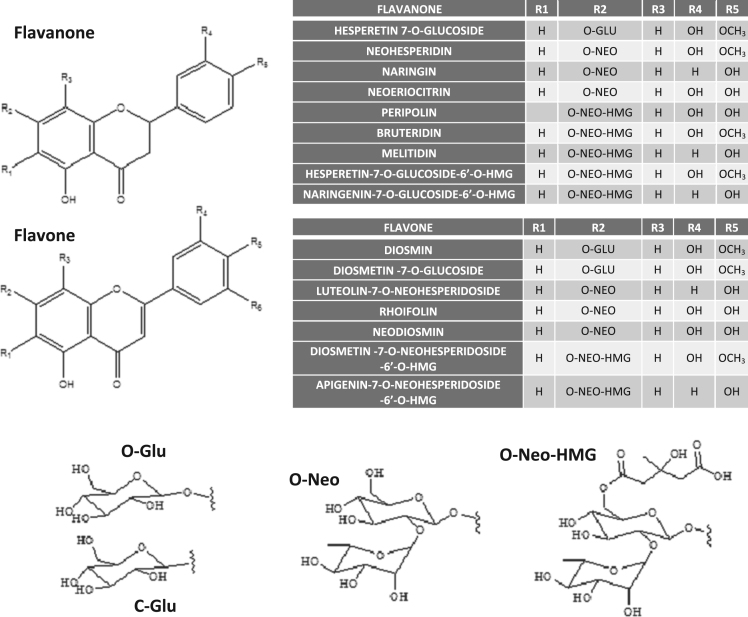
Structures of parent compounds of 6 main aglycones present in A-BPF and their sugar moieties: O-Glu, O-glucoside; C-glucose, C-glucoside; O-Neo, O-Neohesperidoside; O-Neo-HMG, 3-hydroxy-3-methyl-glutaryl-neohesperidoside.

Bergamot Polyphenol Fraction (BPF®) is a natural mixture of *Citrus* flavonoids extracted from processed bergamot fruits [2], [3]. It has been shown to counteract cardiovascular risk factors and to prevent liver steatosis in rats and patients [4], [5], [6], [7], [8], [9], [10]. Protective effects of BPF correlate with its ability to stimulate autophagy in livers of rats fed cafeteria diet [9]. The proautophagic activity of BPF is mediated by a hydrophobic fraction of hydrolysed BPF (A-BPF) containing mainly flavonoid aglycones [1]. Table 1 reports the LC-HRMS output parameters for six major flavonoid aglycones identified in A-BPF, such as retention times (RT), theoretical and measured mass to charge ratio (*m*/*z*), signal intensity as well as a calculated relative and absolute abundance of each flavonoid in the mix (see material and methods). The aglycones listed in Table 1 (column A) originate from several known and unknown parent compounds (i.e. flavonoid glycosides), shown in Fig. 1, that have been previously identified among bergamot polyphenols [3].

**Table 1:** Output parameters of LC-HRMS analysis of flavonoid aglycones identified in A-BPF and their expected parent compounds. Column G shows a theoretical quantitative representation of aglycones in A-BPF, while the H column the amount (in μg) of aglycones in 60 μg of A-BPF, as calculated based on data in G.

**A**	**B**	**C**	**D**	**E**	**F**	**G**	**H**	**I**
**Name**	**RT (MIN)**	**Molecular formula**	**m/z [M−H]**^**−**^**(measured)**	***m*/*z* [M−H]**^−^**(theoretical)**	**Signal intensity (NL) x E6**	**% Total signal**	**in 60 μg**	**Parent compounds**
**ERIODICTYOL**	21.09	C_15_H_12_O_6_	287.0564	287.0561	34.6	20.51	12.3	NEOERIOCITRIN, ERIODICTYOL-7-O-NEOHESPERIDOSIDE-6′′-O-HMG (PERIPOLINA)
**NARINGENIN**	23.61	C_15_H_12_O_5_	261.0615	261.0612	55.9	33.4	19.9	MELITIDIN, NARINGIN
**HESPERETIN**	24.38	C_16_H_14_O_6_	301.0722	301.0718	51.8	30.7	18.4	BRUTERIDIN, HESPERETIN-7-O-GLUCOSIDE, NEOHESPERIDIN
**LUTEOLIN**	24.46	C_15_H_10_O_6_	285.0408	285.0405	3.99	2.37	1.4	LUTEOLIN-7-O-NEOHESPERIDOSIDE
**APIGENIN**	26.47	C_15_H_10_O_5_	269.0459	269.0455	7.01	4.16	2.5	RHOIFOLIN, APIGENIN-7-O-NEOHESPERIDIOSIDE-6′′-O-HMG
**DIOSMETIN**	26.70	C_16_H_12_O_6_	299.0564	299.0561	15.4	9.13	5.5	DIOSMIN, NEODIOSMIN, DIOSMETIN-7-O-GLUCOSIDE, DIOSMETIN-7-O-NEOHESPERIDOSIDE-6′′-O-HMG

**Fig. 2:**
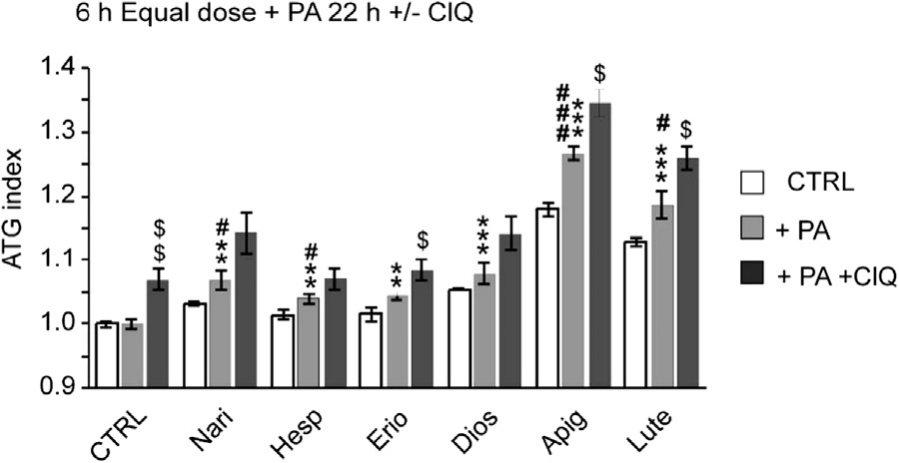
Proautophagic activities of flavonoid aglycones in hepatic cells with high lipid content. ClQ co-treatments further increase ATG index. (A) GR-LC3-HepG2 cells were treated with PA 0,3 mM or vehicle (ethanol, EtOH) for 22 h. Subsequently, the medium was exchanged and aglycones (30 μg/mL) were added for 6 h. 2 h before analysis ClQ (50 μM) or vehicle (H_2_O) was added to PA-treated cells. Red and green fluorescence was recorded by flow cytometry. The graph shows the mean ATG index +/− SEM, from four independent experiments performed each time with triplicate independent samples. Naringenin (Nari), hesperetin (Hesp) and eriodictyol (Erio), followed by diosmetin(Dios), apigenin (Apig) and luteolin (Lute). Red/green ratio data are normalized to controls. Statistical analysis: student *T*-test; *p<0.05, **p<0.01, ***p<0.001, when compared to vehicle-treated controls; ^#^*p*<0.05, ^##^*p*<0.01, ^###^*p*<0.001 when compared to the relative PA-untreated, the same flavonoid-treated cells or ^$^*p*<0.05, ^$$^*p*<0.01, when compared to respective PA-treated, ClQ-untreated cells. See S1 data set (Supplementary data set S1) with row data supporting this figure.

The exposure of hepatocytes to palmitic acid (PA) causes an accumulation of intracellular lipid droplets and models non-alcoholic fatty liver disease (NAFLD) *in vitro* [1]. We used this approach to measure the proautophagic activity of six main flavonoid aglycones present in A-BPF in the presence and absence of lipotoxic stress (Fig. 2). This was done by the qualitative analysis, i.e. equal concentrations of aglycones were used to induce autophagy in HepG2 cells expressing DsRed -LC3- GFP, which turns red when autophagy is induced or LC3-II accumulates [1], [11]. GR-LC3-HepG2 cells were treated with PA (0.3 mM) to cause intracellular lipid overload, and 22 h later exposed to flavonoid aglycons +/− chloroquine (ClQ) to address the autophagic flux modulation. These data were then compared with ATG index (red/green ratio) induced in the absence of PA by six polyphenols in independent experiments performed otherwise under identical conditions (Fig. 2 and Supplementary data set S1).

**Fig. 3:**
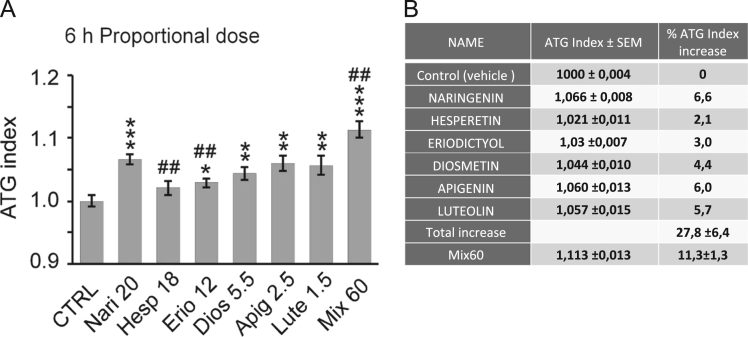
Proportional analysis of proautophagic activities of aglycones present in A-BPF. (A) GR-LC3-HepG2 cells were treated for 6 h with the doses of pure aglycones corresponding to those present in 60 μg A-BPF (reported as numbers of μg/mL after compound abbreviation) or with the mix of these compounds (Mix60) and analysed for ATG index in six independent samples +/− SEM. Statistical analysis: two-tailed, unpaired T-test; *p<0.05, **p<0.01, ***p<0.001 when compared to control (CTRL), vehicle-treated cells; ^#^*p*<0.05, ^##^*p*<0.01 when compared to naringenin. (B) Table showing the analysis of additive effects of six flavonoid aglycones on ATG index. See Supplementary data set S2 (Multimedia Component 3) with row data supporting this figure.

Next, we addressed the quantitative contribution of each aglycone present in A-BPF to the proautophagic activity of A-BPF, which we defined here as “proportional analysis” of proautophagic activity as opposed to “qualitative analysis”, shown in Fig. 2, where equal doses of compounds are compared for their activity. We calculated the *proportional* amounts of six aglycones, as described in Experimental design. Then we treated GR-LC3-HepG2 cells with calculated amounts of standards and analyzed autophagy by flow cytometry 6 h post-treatment. In the same experiment, we also measured the ATG index induced by the mix of all six compounds, used at proportional concentrations to reconstitute 60 μg/mL of A-BPF (Mix60) (Table 1). The ATG index data for six tested aglycones and their Mix are reported in Fig. 3A. The data in Fig. 3A are supported by Supplementary data set S2 presenting the row data used for this analysis. The Excel file S2 contains tables with mean fluorescence intensities values recorded in 54 independent cell samples in two independent experiments. This Excel file also shows how the mean ATG index is calculated and normalized and performs statistical analysis of the data. Examples of flow cytometry dot-plots with raw data and relevant gates used for analysis are also attached to Supplemetary data set S2.

Next, we tried to evaluate if six polyphenols contributed in additive or synergistic fashion to the overall autophagic activity of BPF. The data in Fig. 3B are a numeric representation of data presented in Fig. 3A and they show that the sum of mean ATG index increases caused by individual compounds is higher than the ATG index induced by the Mix of aglycones. These data would rather suggest additive as well competitive effects, rather than synergistic effects of tested flavonoids on autophagy.

## 2 Experimental design, materials and methods

### 2.1 Cell culture

GR-LC3-HepG2 cells were descried previously in Lascala et al. [1]. They were cultured in DMEM complete medium (4.5 g/L glucose) as described for the HepG2 cells [1]. For experiments, the cells were seeded at the density 4×10^4^ cm^−^^2^ and the last medium change was performed 24 h before the end of the experiment.

### 2.2 Reagents

Naringenin, hesperetin, eriodictyol, diosmetin, apigenin and luteolin were purchased from Extrasynthese (Geney Cedex, France) as ≥99% pure (HPLC-grade) powders, which were solubilized in EtOH 100% to stock solutions 5 mg/mL, except for diosmetin and apigenin diluted as 2 mg/mL stock due to their low solubility. BPF® was provided by *Herbal and Antioxidant Derivatives* srl (H&AD srl), Bianco, Italy. A-BPF was prepared by acid hydrolysis of BPF and isolation of hydrophobic phases from crude hydrolysate, according to the procedure described in Lascala et al. [1] “Mix60”, or “Mix”, was prepared by mixing ethanol solutions of six aglycones (as above) in natural proportions as found in A-BPF, according to the data presented in Table 1, column G and H. ChlQ (25 mM stock in PBS), PA (0.1 to 1 M stock in EtOH) were from Sigma-Aldrich. These compounds were kept in aliquots at −80 °C or −20 °C (shorter storage) and thawed shortly before each treatment.

### 2.3 Production of retroviruses coding for DsRed-LC3-GFP

To produce GR-LC3-HepG2 cells stably expressing DsRed-LC3-GFP reporter, recombinant retroviruses coding were generated and used to infect HepG2 cells. To produce viral stocks, HEK 293 T cells (one 100 mm plate at 90–95% confluence) were transfected with pUMVC (10 μg), pCMV-VSV- G (4 μg) and pQCXI-Puro-DsRed-LC3-GFP (10 μg) from Addgene (Cambridge, MA, USA), using Lipofectamine 2000 (Life Tech., Invitrogen, 11668027) according to manufacturer׳s instructions. After an overnight incubation, cell medium was replaced with 5 mL of RPMI medium (Life Tech., Invitrogen, 11875093), supplemented with 2% FBS. 48 h post-transfection, cell supernatant was collected, filtered through a 0.45 μm filter membrane, and supplemented with FBS (10% final concentration). Prior to viral transduction, HepG2 cells were seeded at a concentration of 4×10^5^/well in six-well plates, and 5 mL of viral supernatant was collected and used to infect cells by spinoculation in 6-well plate sealed with parafilm, at 720 g, *T*=32 °C, in presence of polybrene (8 μg/mL, Sigma-Aldrich, 107689) for 50 min. After 4 h of incubation at 37 °C, 5% CO_2_, cells were washed with PBS and switched to standard medium. At 48 h post-infection, puromycin (2 μg/mL, Sigma-Aldrich, P8833) was added for 8 days. The transduction efficiency was evaluated by FACS analysis as the EGFP-positive cell fraction. To maintain high expression of DsRed-LC3-GFP the cells were cultivated in DMEM complete supplemented with puromycin (1 μg/mL). For experiments cells were plated without puromycin.

### 2.4 Flow cytometry analysis of autophagy

GR-LC3-HepG2 cells were seeded on 24-well plates and cultured as described in *Cell culture* section above. After 3 days pre-treatments with PA (Sigma-Aldrich, 0,3 mM final, 150 mM stock in EtOH) were performed 22–24 h before medium change and addition of flavonoid aglycones or other substances 6 h before cell harvesting. ClQ (50 μM in H2O) was added 2 h before cell harvesting. For the treatments all wells were treated with the same volume of EtOH (usually 3 μL), DMSO and water, which were used as vehicles. Each treatment series were performed in triplicate, but at different times. 6 h later the cells were washed once in PBS and collected by trypsinization as described before [12]. Briefly, pelleted cells were resuspended in 0.45 mL PBS containing 1% FBS and 0.1 mM EDTA (Sigma-Aldrich, E5134). To exclude dead cells in analysis they were treated with 75 μl of trypan blue (TB) solution (0.008% in PBS), added 60 s before flow cytometry recording. This was not necessary for experiments with low cell mortality (below 5% in all samples). However, the addition of TB did not influence significantly the ATG index. Cells were acquired in 502 nm (FITC, green), 556 nm (PE, red) and 655 (PerCP-Cy5, blue) channels by FACSCanto II (BD Biosciences, Erenbodgem, Belgium). Populations of interest were identified: single cell population (or P1), viable single cells (or P6) and GFP and Ds-Red/GFP positive population (indicated as Q2). See row data sets S1 and S2. Mean fluorescence intensity (MFI) for red and green channels was determined in the populations of interest by BD FACSDiva software. The autophagy index (ATG index) was calculated as the ratio of red to green channel MFI in triplicate samples for each experimental point for a populations of interest (P6 or Q2). The data were normalized to the mean ATG index of three control samples. For further details see examples of ATG index analysis in Excel files provided as in Supplementary data sets S1 and S2.

### 2.5 A-BPF preparation and analysis

A-BPF was obtained from BPF® by acid hydrolysis, as reported in Lascala et al. [1]. Subsequently, 2 mg of A-BPF were used to prepare a sample for LC-HRMS analysis according to the procedures described in the companion paper [1]. BPF® is a kind gift of the owner of the BPF trademark, Herbal and Antioxidant Derivatives S.r.l. (H&AD S.r.l.), Bianco (RC), Italy.

### 2.6 Mass spectrometry

Q-ExactiveTM (Thermo Scientific) mass spectrometer was operated using electrospray with negative polarities at 35,000 resolving power (defined as FWHM at *m*/*z* 200), IT 150 ms, and ACG target=1,000,000, in full scan analysis (mass range 140–900 amu). Source conditions were: spray voltage 2.9 KV, sheath gas: 30, arbitrary units, Auxiliary gas: 10, probe heater temperature: 280 °C; capillary temperature: 320 °C; S-Lens RF Level: 50. The instrument was calibrated by Thermo calibration solutions prior to the beginning the analysis.

### 2.7 *Proportional* analysis of activity of individual compounds present A-BPF phytocomplex

To perform a *proportional* analysis, we estimated the amounts of six major flavonoids present in 60 µg/mL of A-BPF based on LC–mass spectrometry data described in Table 1. To this end, we assumed that the ion current signal intensity (SI) is proportional to the relative quantity of each flavonoid, which is well applicable to structurally similar compounds, according to our previous observations [13]. For sake of simplicity, we assumed that the total quantity of six identified polyphenols corresponds to 100% of A-BPF and 100% of total ion current signal intensity (TSI). SI for each flavonoid was divided by TSI and multiplied by 60 µg/mL to calculated the *proportional* amounts flavonoids contributing to A-BPF phytocomplex.

## Footnotes

Appendix A Supplementary data associated with this article can be found in the online version at doi:10.1016/j.dib.2018.05.139.
