# Supplementary material for: Qualitative and quantitative analysis of the proautophagic activity of Citrus flavonoids from Bergamot Polyphenol Fraction
Source: Data Brief. 2018 May 31;19:1327–34. doi: 10.1016/j.dib.2018.05.139 (PMC6140830; doi:10.1016/j.dib.2018.05.139)
Supplement: Supplementary file 1 — Supplementary material [file mmc1.pdf]

UNIVERSITÀ DEGLI STUDI MAGNA GRÆCIA
DI CATANZARO
Dipartimento di Scienze della Salute

NO CONFLICTS OF INTEREST DECLARATION

DIB-D-18-01022: **“Qualitative and quantitative analysis of the proautophagic activity of Citrus flavonoids from Bergamot Polyphenol Fraction”** by Janda et al.

The research presented in DIB-D-18-01022 Ms. received a small financial support from Herbal and Antioxidant Derivatives (H&AD) srl company, which is the company producing BPF®, as stated in *Acknowledgments* section of this data article.

Nevertheless, I wish to declare that the company had absolutely NO influence on the scientific content of this work and thus the research relative to this data article was conducted in a fully independent manner.

Furthermore, I confirm that the manuscript has been read and approved by all named authors and that there are no other persons who satisfied the criteria for authorship, but are not listed.

Signed by the corresponding author.

Elzbieta Janda, UMG, Catanzaro, Italy

Elzbieta Janda, PhD
Department of Health Sciences,
University “Magna Graecia”,
viale Europa, Campus Germaneto
88100 Catanzaro, Italy.
e-mail: janda@unicz.it
phone +39 09613694143
cell. +39 366 6215 493
