# Supplementary material for: Qualitative and quantitative analysis of the proautophagic activity of Citrus flavonoids from Bergamot Polyphenol Fraction
Source: Data Brief. 2018 May 31;19:1327–34. doi: 10.1016/j.dib.2018.05.139 (PMC6140830; doi:10.1016/j.dib.2018.05.139)
Supplement: Supplementary file 4 — Supplementary material [file mmc4.pdf]

FACSDiva Version 6.1.2

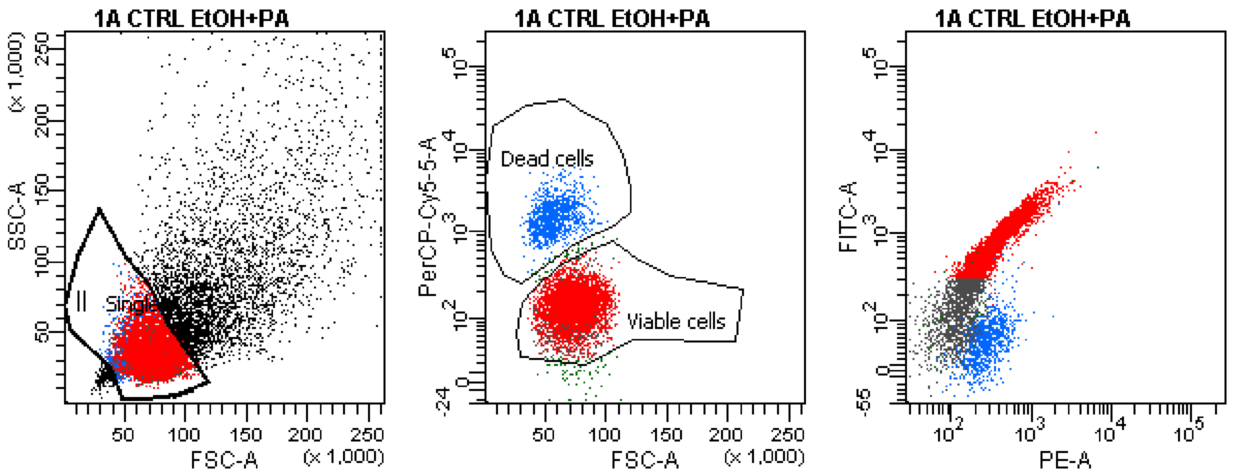

Tube: 1A CTRL EtOH+PA			
**Population**	**#Events**	**%Parent**	**%Total**
All Events	10,000	####	100.0
Singlets	5,953	59.5	59.5
Dead cells	1,042	17.5	10.4
Viable cells	4,807	80.7	48.1
Q1	14	0.3	0.1
Q2	3,539	73.6	35.4
Q3	445	9.3	4.4
Q4	809	16.8	8.1
P1	1,313	27.3	13.1
NOT(P1)	3,494	72.7	34.9

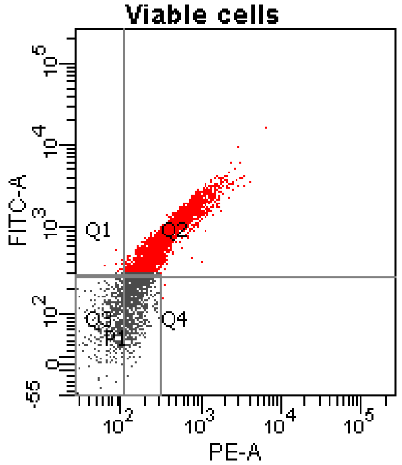

Tube Name: 1A CTRL EtOH+PA

Population	#Events	%Parent	FITC-A Mean	PE-A Mean
Singlets	5,953	59.5	601	363
Dead cells	1,042	17.5	64	290
Viable cells	4,807	80.7	719	379
Q1	14	0.3	337	92
Q2	3,539	73.6	927	469
Q3	445	9.3	90	75
Q4	809	16.8	160	159
P1	1,313	27.3	142	131
NOT(P1)	3,494	72.7	936	472
