# Supplementary material for: Qualitative and quantitative analysis of the proautophagic activity of Citrus flavonoids from Bergamot Polyphenol Fraction
Source: Data Brief. 2018 May 31;19:1327–34. doi: 10.1016/j.dib.2018.05.139 (PMC6140830; doi:10.1016/j.dib.2018.05.139)
Supplement: Supplementary file 5 — Supplementary material [file mmc5.pdf]

FACSDiva Version 6.1.2

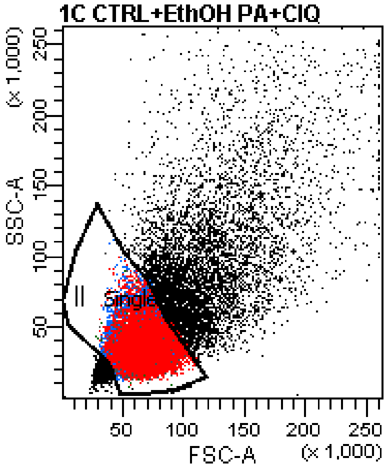

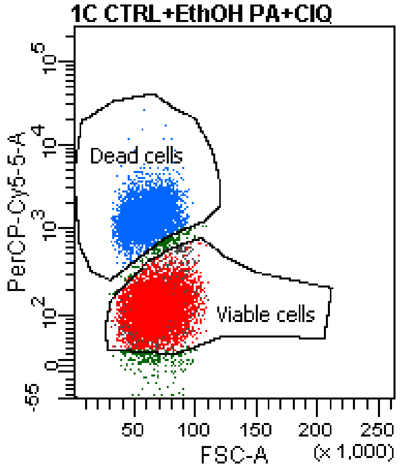

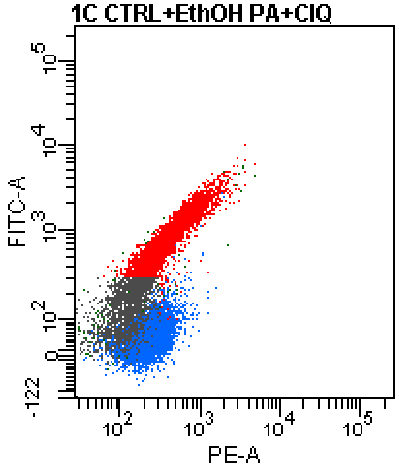

Tube: 1C CTRL+EthOH PA+CIQ

Population	#Events	%Parent	%Total
All Events	20,000	####	100.0
Singlets	12,624	63.1	63.1
Dead cells	5,300	42.0	26.5
Viable cells	6,923	54.8	34.6
Q1	25	0.4	0.1
Q2	5,055	73.0	25.3
Q3	660	9.5	3.3
Q4	1,183	17.1	5.9
P1	1,912	27.6	9.6
NOT(P1)	5,011	72.4	25.1

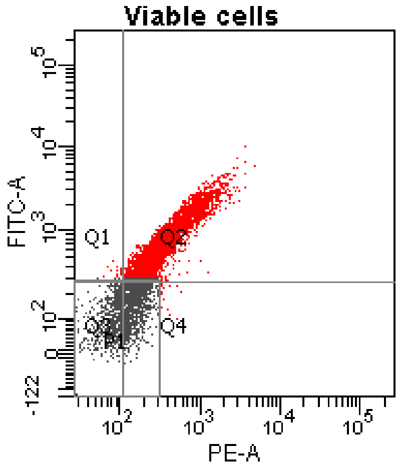

Tube Name: 1C CTRL+EthOH PA+CIQ

Population	#Events	%Parent	FITC-A Mean	PE-A Mean
Singlets	12,624	63.1	418	328
Dead cells	5,300	42.0	45	242
Viable cells	6,923	54.8	705	396
Q1	25	0.4	318	93
Q2	5,055	73.0	914	493
Q3	660	9.5	91	76
Q4	1,183	17.1	164	166
P1	1,912	27.6	144	133
NOT(P1)	5,011	72.4	919	496
