# Supplementary material for: Qualitative and quantitative analysis of the proautophagic activity of Citrus flavonoids from Bergamot Polyphenol Fraction
Source: Data Brief. 2018 May 31;19:1327–34. doi: 10.1016/j.dib.2018.05.139 (PMC6140830; doi:10.1016/j.dib.2018.05.139)
Supplement: Supplementary file 6 — Supplementary material [file mmc6.pdf]

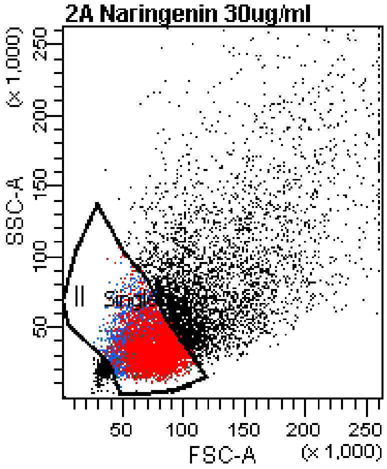

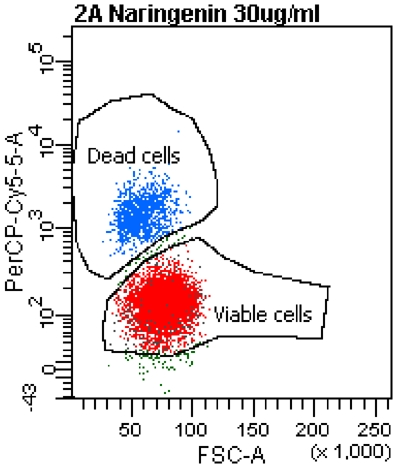

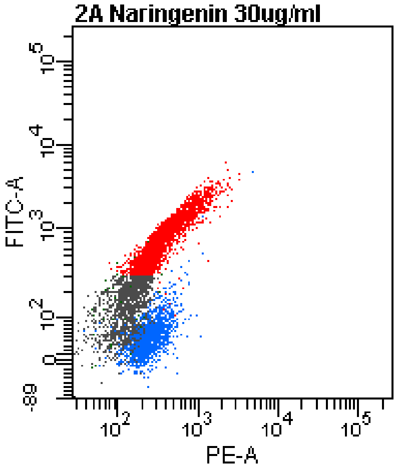

Tube: 2A Naringenin 30ug/ml

Population	#Events	%Parent	%Total
All Events	10,000	####	100.0
Singlets	5,731	57.3	57.3
Dead cells	1,342	23.4	13.4
Viable cells	4,274	74.6	42.7
Q1	12	0.3	0.1
Q2	3,126	73.1	31.3
Q3	360	8.4	3.6
Q4	776	18.2	7.8
P1	1,190	27.8	11.9
NOT(P1)	3,084	72.2	30.8

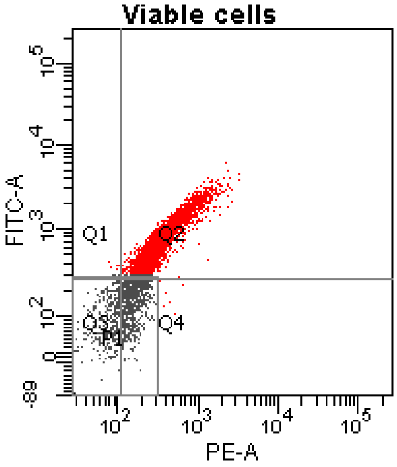

Tube Name: 2A Naringenin 30ug/ml

Population	#Events	%Parent	FITC-A Mean	PE-A Mean
Singlets	5,731	57.3	521	342
Dead cells	1,342	23.4	55	264
Viable cells	4,274	74.6	671	369
Q1	12	0.3	315	95
Q2	3,126	73.1	866	455
Q3	360	8.4	90	77
Q4	776	18.2	160	165
P1	1,190	27.8	145	137
NOT(P1)	3,084	72.2	873	459
