# Supplementary material for: Qualitative and quantitative analysis of the proautophagic activity of Citrus flavonoids from Bergamot Polyphenol Fraction
Source: Data Brief. 2018 May 31;19:1327–34. doi: 10.1016/j.dib.2018.05.139 (PMC6140830; doi:10.1016/j.dib.2018.05.139)
Supplement: Supplementary file 7 — Supplementary material [file mmc7.pdf]

FACSDiva Version 6.1.2

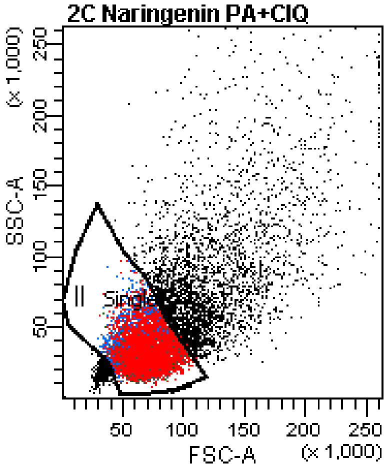

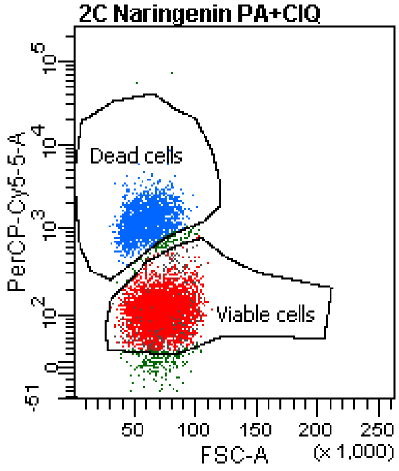

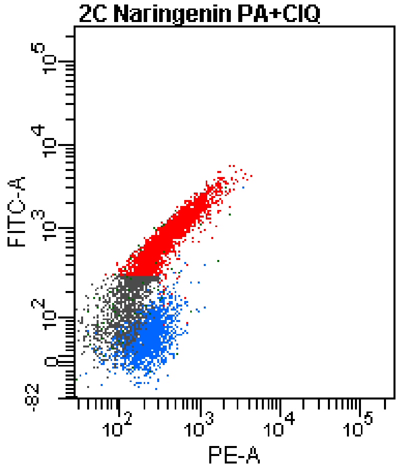

Tube: 2C Naringenin PA+ClQ

Population	#Events	%Parent	%Total
All Events	10,000	####	100.0
Singlets	6,467	64.7	64.7
Dead cells	2,012	31.1	20.1
Viable cells	4,157	64.3	41.6
Q1	25	0.6	0.2
Q2	2,993	72.0	29.9
Q3	449	10.8	4.5
Q4	690	16.6	6.9
P1	1,191	28.7	11.9
NOT(P1)	2,966	71.3	29.7

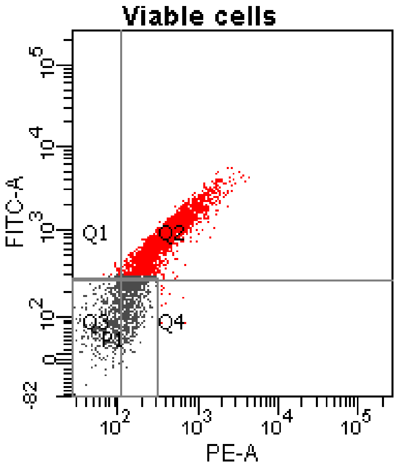

Tube Name: 2C Naringenin PA+ClQ

Population	#Events	%Parent	FITC-A Mean	PE-A Mean
Singlets	6,467	64.7	453	323
Dead cells	2,012	31.1	50	236
Viable cells	4,157	64.3	657	371
Q1	25	0.6	308	97
Q2	2,993	72.0	857	467
Q3	449	10.8	94	73
Q4	690	16.6	166	160
P1	1,191	28.7	145	125
NOT(P1)	2,966	71.3	862	470
