# Supplementary material for: Qualitative and quantitative analysis of the proautophagic activity of Citrus flavonoids from Bergamot Polyphenol Fraction
Source: Data Brief. 2018 May 31;19:1327–34. doi: 10.1016/j.dib.2018.05.139 (PMC6140830; doi:10.1016/j.dib.2018.05.139)
Supplement: Supplementary file 8 — Supplementary material [file mmc8.pdf]

FACSDiva Version 6.1.2

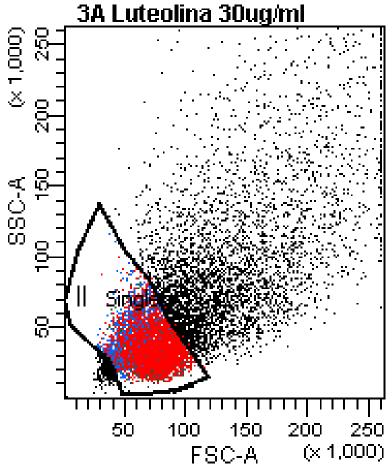

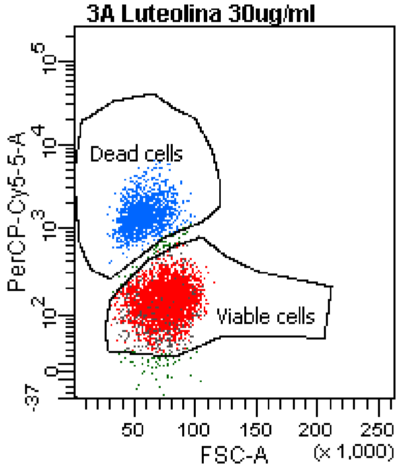

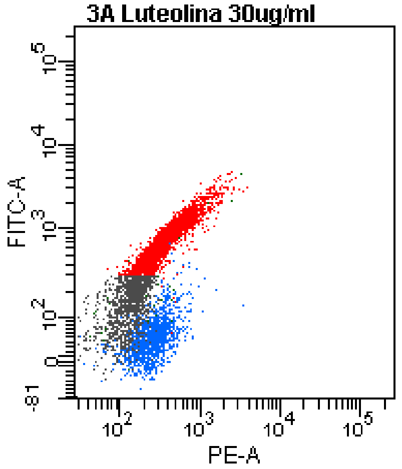

Tube: 3A Luteolina 30ug/ml

Population	#Events	%Parent	%Total
All Events	10,000	####	100.0
Singlets	5,351	53.5	53.5
Dead cells	1,642	30.7	16.4
Viable cells	3,597	67.2	36.0
Q1	11	0.3	0.1
Q2	2,479	68.9	24.8
Q3	355	9.9	3.6
Q4	752	20.9	7.5
P1	1,170	32.5	11.7
NOT(P1)	2,427	67.5	24.3

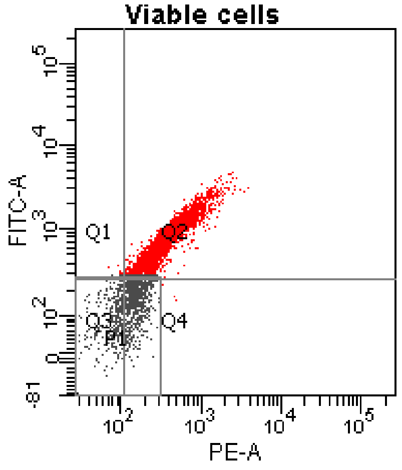

Tube Name: 3A Luteolina 30ug/ml

Population	#Events	%Parent	FITC-A Mean	PE-A Mean
Singlets	5,351	53.5	430	334
Dead cells	1,642	30.7	50	272
Viable cells	3,597	67.2	604	362
Q1	11	0.3	300	96
Q2	2,479	68.9	812	465
Q3	355	9.9	94	77
Q4	752	20.9	162	162
P1	1,170	32.5	148	137
NOT(P1)	2,427	67.5	824	471
