# Supplementary material for: Qualitative and quantitative analysis of the proautophagic activity of Citrus flavonoids from Bergamot Polyphenol Fraction
Source: Data Brief. 2018 May 31;19:1327–34. doi: 10.1016/j.dib.2018.05.139 (PMC6140830; doi:10.1016/j.dib.2018.05.139)
Supplement: Supplementary file 9 — Supplementary material [file mmc9.pdf]

FACSDiva Version 6.1.2

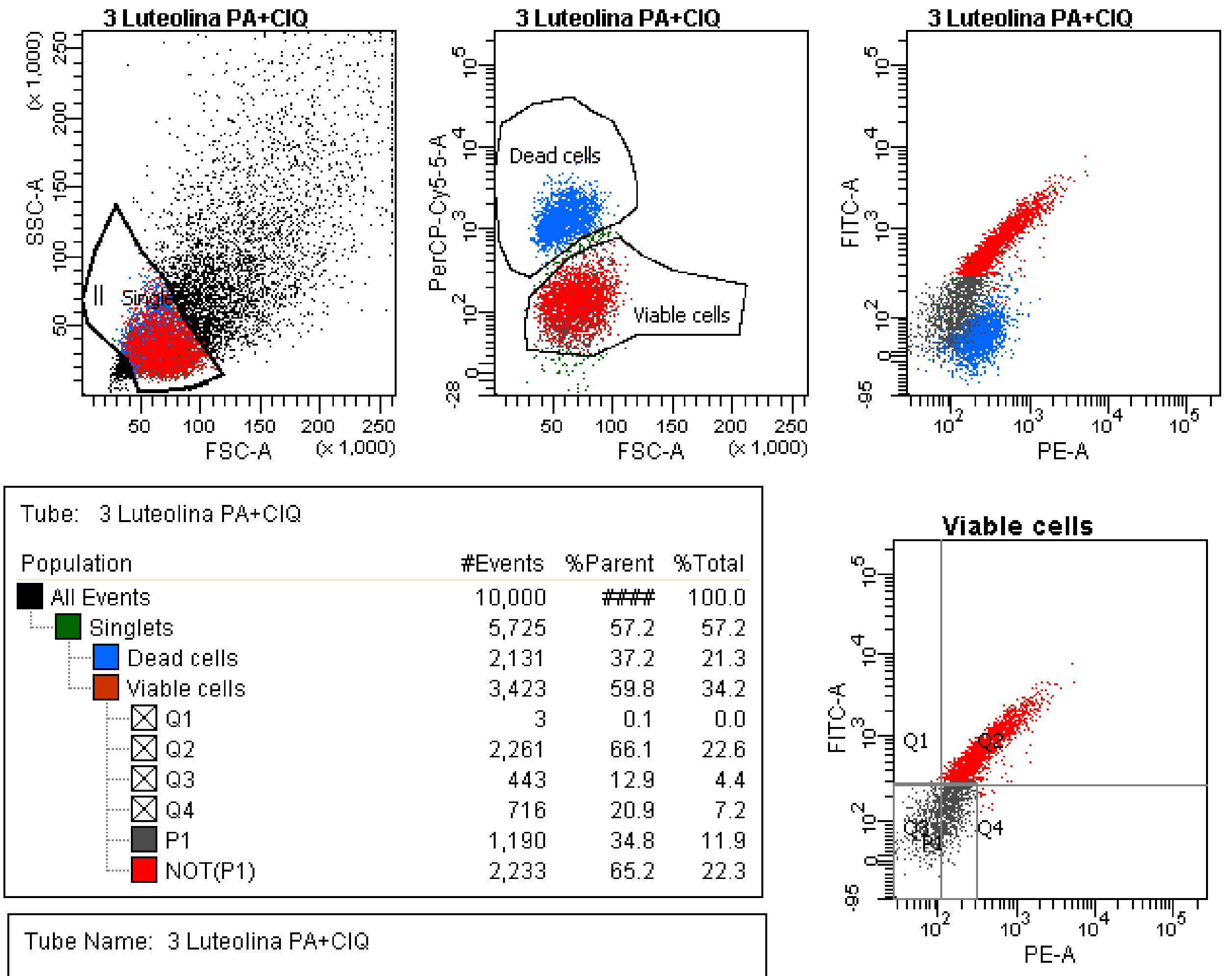

Tube: 3 Luteolina PA+ClQ

Population	#Events	%Parent	%Total
All Events	10,000	####	100.0
Singlets	5,725	57.2	57.2
Dead cells	2,131	37.2	21.3
Viable cells	3,423	59.8	34.2
Q1	3	0.1	0.0
Q2	2,261	66.1	22.6
Q3	443	12.9	4.4
Q4	716	20.9	7.2
P1	1,190	34.8	11.9
NOT(P1)	2,233	65.2	22.3

Tube Name: 3 Luteolina PA+ClQ

Population	#Events	%Parent	FITC-A Mean	PE-A Mean
Singlets	5,725	57.2	372	321
Dead cells	2,131	37.2	47	245
Viable cells	3,423	59.8	573	368
Q1	3	0.1	297	93
Q2	2,261	66.1	797	489
Q3	443	12.9	90	74
Q4	716	20.9	167	167
P1	1,190	34.8	143	130
NOT(P1)	2,233	65.2	803	495
