# Supplementary material for: Qualitative and quantitative analysis of the proautophagic activity of Citrus flavonoids from Bergamot Polyphenol Fraction
Source: Data Brief. 2018 May 31;19:1327–34. doi: 10.1016/j.dib.2018.05.139 (PMC6140830; doi:10.1016/j.dib.2018.05.139)
Supplement: Supplementary file 10 — Supplementary material [file mmc10.pdf]

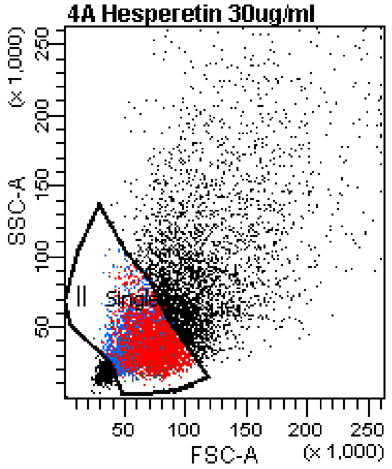

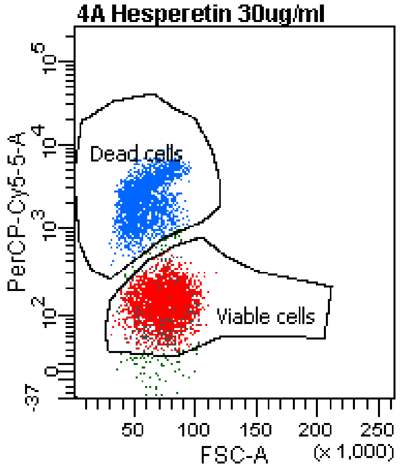

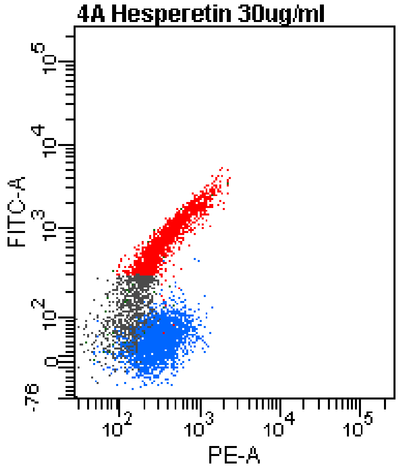

Tube: 4A Hesperetin 30ug/ml			
Population	#Events	%Parent	%Total
All Events	10,000	####	100.0
Singlets	5,840	58.4	58.4
Dead cells	2,650	45.4	26.5
Viable cells	3,083	52.8	30.8
Q1	8	0.3	0.1
Q2	2,220	72.0	22.2
Q3	243	7.9	2.4
Q4	612	19.9	6.1
P1	886	28.7	8.9
NOT(P1)	2,197	71.3	22.0

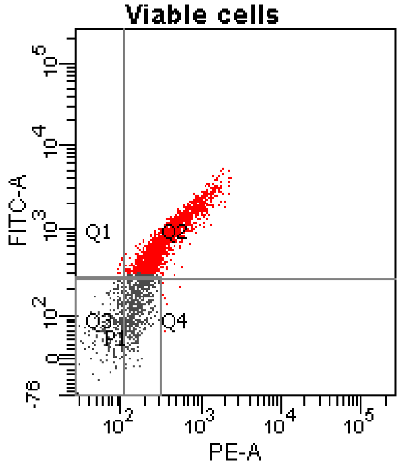

Tube Name: 4A Hesperetin 30ug/ml

Population	#Events	%Parent	FITC-A Mean	PE-A Mean
Singlets	5,840	58.4	371	345
Dead cells	2,650	45.4	42	323
Viable cells	3,083	52.8	653	366
Q1	8	0.3	341	97
Q2	2,220	72.0	854	452
Q3	243	7.9	85	76
Q4	612	19.9	154	171
P1	886	28.7	140	144
NOT(P1)	2,197	71.3	860	456
