# Supplementary material for: Qualitative and quantitative analysis of the proautophagic activity of Citrus flavonoids from Bergamot Polyphenol Fraction
Source: Data Brief. 2018 May 31;19:1327–34. doi: 10.1016/j.dib.2018.05.139 (PMC6140830; doi:10.1016/j.dib.2018.05.139)
Supplement: Supplementary file 11 — Supplementary material [file mmc11.pdf]

FACSDiva Version 6.1.2

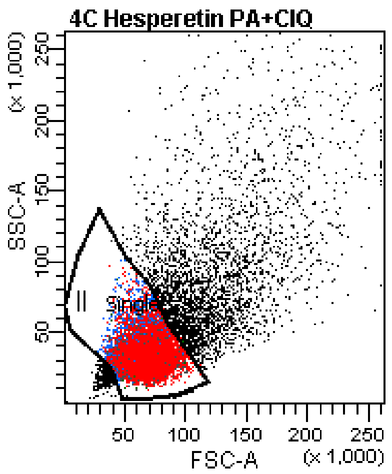

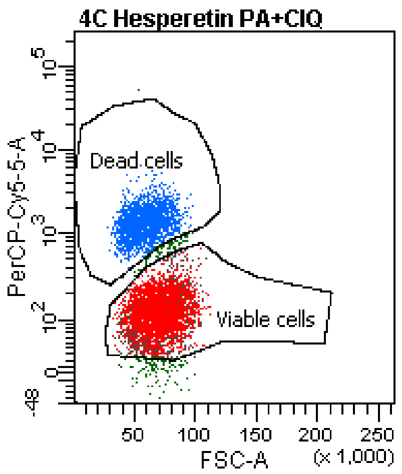

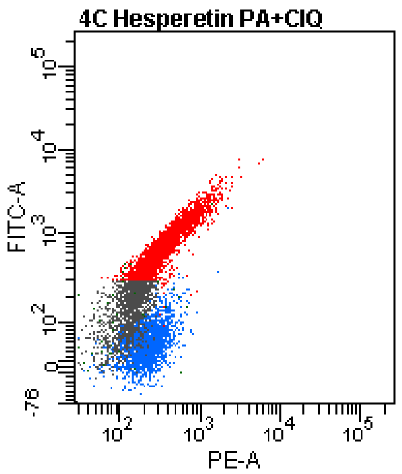

Tube: 4C Hesperetin PA+ClQ

Population	#Events	%Parent	%Total
All Events	10,000	####	100.0
Singlets	6,459	64.6	64.6
Dead cells	2,048	31.7	20.5
Viable cells	4,195	64.9	41.9
Q1	16	0.4	0.2
Q2	2,988	71.2	29.9
Q3	372	8.9	3.7
Q4	819	19.5	8.2
P1	1,259	30.0	12.6
NOT(P1)	2,936	70.0	29.4

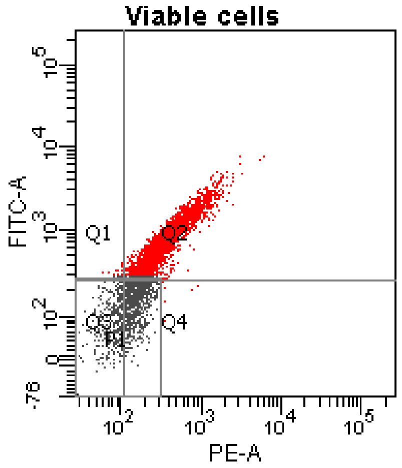

Tube Name: 4C Hesperetin PA+ClQ

Population	#Events	%Parent	FITC-A Mean	PE-A Mean
Singlets	6,459	64.6	461	328
Dead cells	2,048	31.7	50	236
Viable cells	4,195	64.9	669	378
Q1	16	0.4	307	90
Q2	2,988	71.2	882	475
Q3	372	8.9	90	75
Q4	819	19.5	163	166
P1	1,259	30.0	148	138
NOT(P1)	2,936	70.0	893	480
