# Supplementary material for: Qualitative and quantitative analysis of the proautophagic activity of Citrus flavonoids from Bergamot Polyphenol Fraction
Source: Data Brief. 2018 May 31;19:1327–34. doi: 10.1016/j.dib.2018.05.139 (PMC6140830; doi:10.1016/j.dib.2018.05.139)
Supplement: Supplementary file 12 — Supplementary material [file mmc12.pdf]

FACSDiva Version 6.1.2

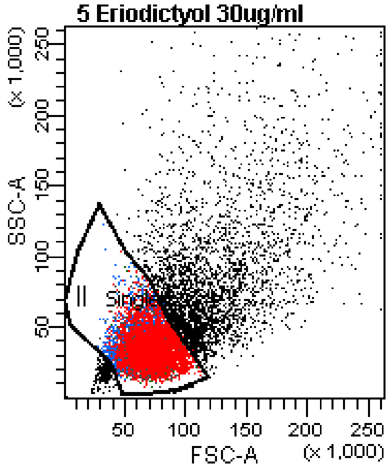

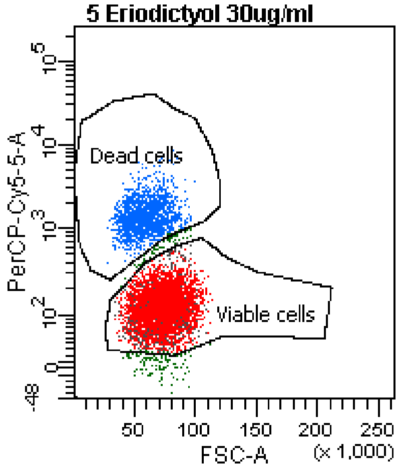

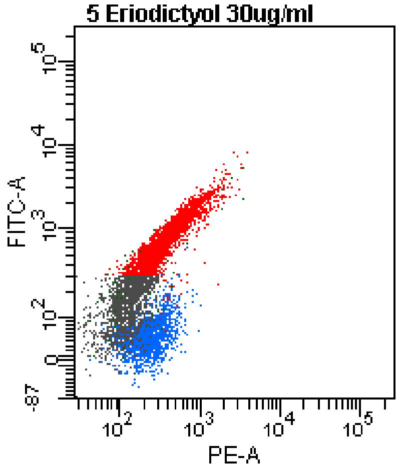

Tube: 5 Eriodictyol 30ug/ml

Population	#Events	%Parent	%Total
All Events	10,000	####	100.0
Singlets	6,429	64.3	64.3
Dead cells	1,596	24.8	16.0
Viable cells	4,623	71.9	46.2
Q1	15	0.3	0.2
Q2	3,251	70.3	32.5
Q3	468	10.1	4.7
Q4	889	19.2	8.9
P1	1,400	30.3	14.0
NOT(P1)	3,223	69.7	32.2

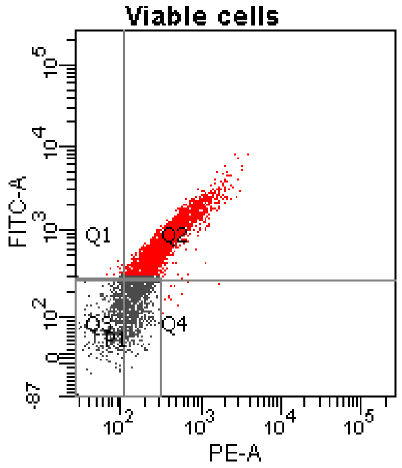

Tube Name: 5 Eriodictyol 30ug/ml

Population	#Events	%Parent	FITC-A Mean	PE-A Mean
Singlets	6,429	64.3	477	327
Dead cells	1,596	24.8	50	259
Viable cells	4,623	71.9	630	354
Q1	15	0.3	313	89
Q2	3,251	70.3	836	446
Q3	468	10.1	97	76
Q4	889	19.2	162	167
P1	1,400	30.3	145	133
NOT(P1)	3,223	69.7	841	450
