# Supplementary material for: Qualitative and quantitative analysis of the proautophagic activity of Citrus flavonoids from Bergamot Polyphenol Fraction
Source: Data Brief. 2018 May 31;19:1327–34. doi: 10.1016/j.dib.2018.05.139 (PMC6140830; doi:10.1016/j.dib.2018.05.139)
Supplement: Supplementary file 13 — Supplementary material [file mmc13.pdf]

# FACSDiva Version 6.1.2

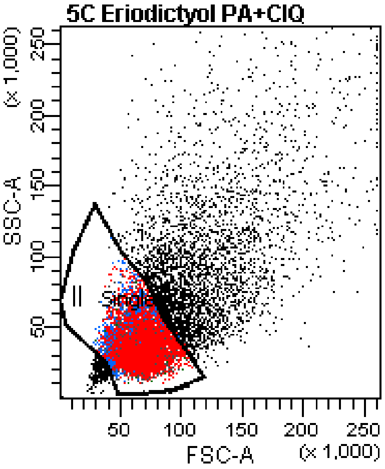


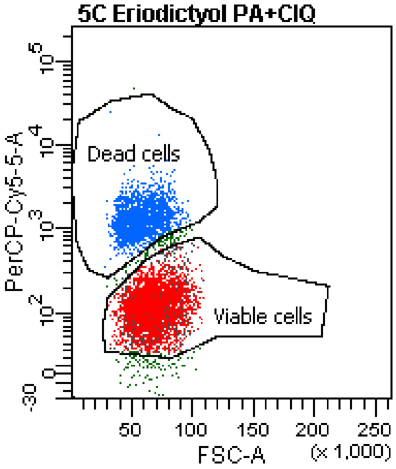


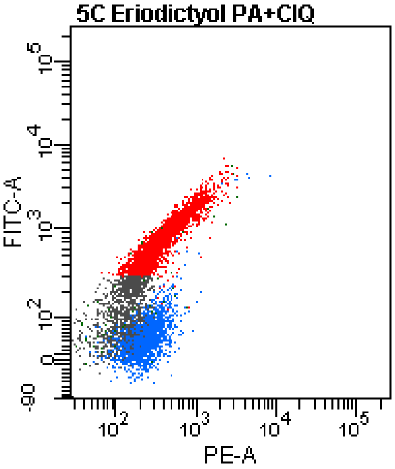


Tube: 5C Eriodictyol PA+CIQ

| Population | #Events | %Parent | %Total |
|---|---|---|---|
| All Events | 10,000 | #### | 100.0 |
| Singlets | 6,136 | 61.4 | 61.4 |
| Dead cells | 2,204 | 35.9 | 22.0 |
| Viable cells | 3,706 | 60.4 | 37.1 |
| Q1 | 7 | 0.2 | 0.1 |
| Q2 | 2,711 | 73.2 | 27.1 |
| Q3 | 368 | 9.9 | 3.7 |
| Q4 | 620 | 16.7 | 6.2 |
| P1 | 1,031 | 27.8 | 10.3 |
| NOT(P1) | 2,675 | 72.2 | 26.8 |

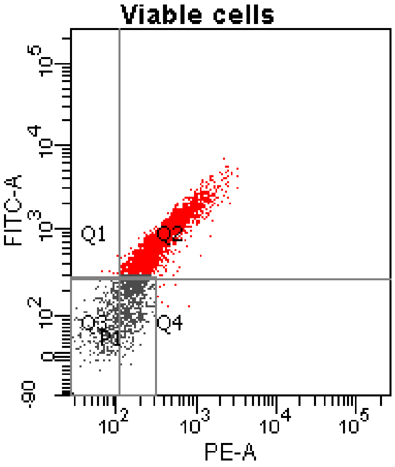


Tube Name: 5C Eriodictyol PA+CIQ

| Population | #Events | %Parent | FITC-A Mean | PE-A Mean |
|---|---|---|---|---|
| Singlets | 6,136 | 61.4 | 449 | 335 |
| Dead cells | 2,204 | 35.9 | 55 | 250 |
| Viable cells | 3,706 | 60.4 | 687 | 387 |
| Q1 | 7 | 0.2 | 336 | 94 |
| Q2 | 2,711 | 73.2 | 891 | 481 |
| Q3 | 368 | 9.9 | 83 | 73 |
| Q4 | 620 | 16.7 | 158 | 166 |
| P1 | 1,031 | 27.8 | 137 | 131 |
| NOT(P1) | 2,675 | 72.2 | 899 | 486 |
