# Supplementary material for: Qualitative and quantitative analysis of the proautophagic activity of Citrus flavonoids from Bergamot Polyphenol Fraction
Source: Data Brief. 2018 May 31;19:1327–34. doi: 10.1016/j.dib.2018.05.139 (PMC6140830; doi:10.1016/j.dib.2018.05.139)
Supplement: Supplementary file 14 — Supplementary material [file mmc14.pdf]

FACSDiva Version 6.1.2

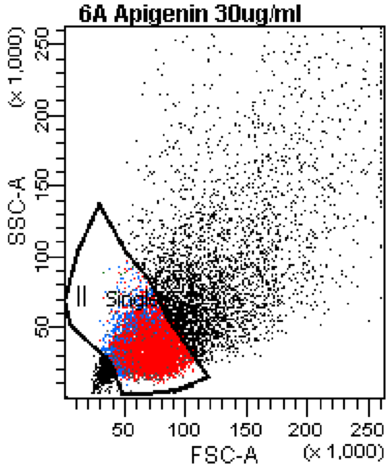

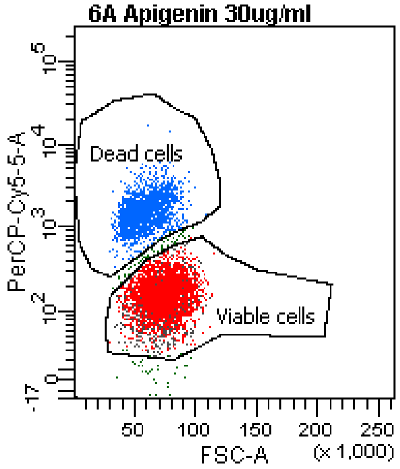

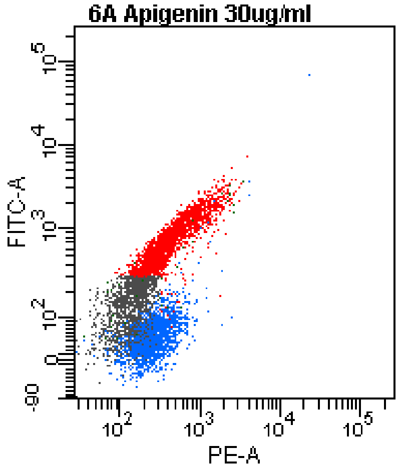

Tube: 6A Apigenin 30ug/ml

Population	#Events	%Parent	%Total
All Events	10,000	####	100.0
Singlets	6,065	60.7	60.7
Dead cells	2,140	35.3	21.4
Viable cells	3,819	63.0	38.2
Q1	8	0.2	0.1
Q2	2,626	68.8	26.3
Q3	335	8.8	3.4
Q4	850	22.3	8.5
P1	1,240	32.5	12.4
NOT(P1)	2,579	67.5	25.8

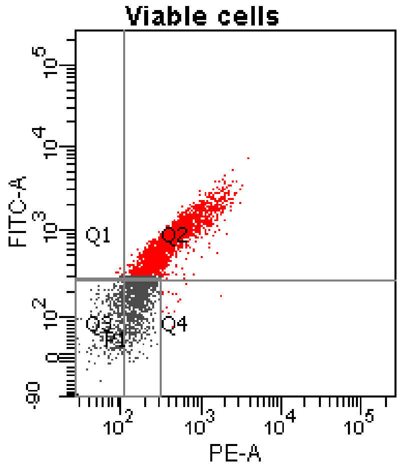

Tube Name: 6A Apigenin 30ug/ml

Population	#Events	%Parent	FITC-A Mean	PE-A Mean
Singlets	6,065	60.7	412	368
Dead cells	2,140	35.3	84	287
Viable cells	3,819	63.0	594	411
Q1	8	0.2	302	95
Q2	2,626	68.8	799	530
Q3	335	8.8	91	78
Q4	850	22.3	163	177
P1	1,240	32.5	150	145
NOT(P1)	2,579	67.5	808	539
