# Supplementary material for: Qualitative and quantitative analysis of the proautophagic activity of Citrus flavonoids from Bergamot Polyphenol Fraction
Source: Data Brief. 2018 May 31;19:1327–34. doi: 10.1016/j.dib.2018.05.139 (PMC6140830; doi:10.1016/j.dib.2018.05.139)
Supplement: Supplementary file 15 — Supplementary material [file mmc15.pdf]

FACSDiva Version 6.1.2

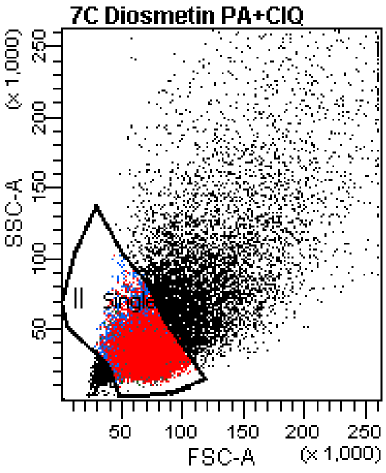

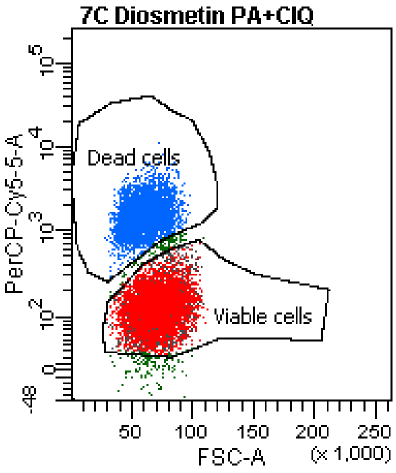

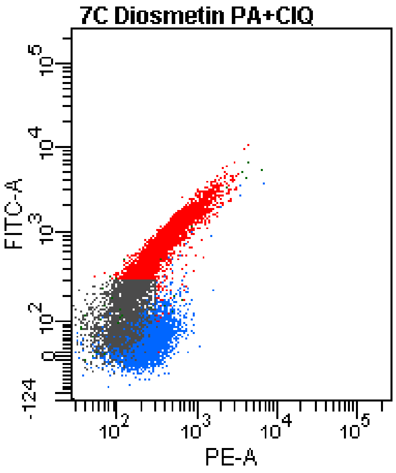

Tube: 7C Diosmetin PA+CIQ

Population	#Events	%Parent	%Total
All Events	20,000	####	100.0
Singlets	12,095	60.5	60.5
Dead cells	5,028	41.6	25.1
Viable cells	6,766	55.9	33.8
Q1	22	0.3	0.1
Q2	4,665	68.9	23.3
Q3	755	11.2	3.8
Q4	1,324	19.6	6.6
P1	2,167	32.0	10.8
NOT(P1)	4,599	68.0	23.0

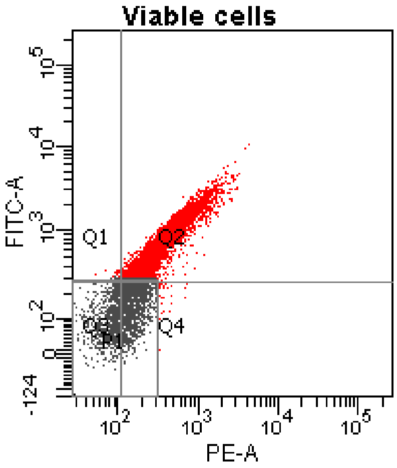

Tube Name: 7C Diosmetin PA+CIQ

Population	#Events	%Parent	FITC-A Mean	PE-A Mean
Singlets	12,095	60.5	384	317
Dead cells	5,028	41.6	48	244
Viable cells	6,766	55.9	635	372
Q1	22	0.3	304	94
Q2	4,665	68.9	860	479
Q3	755	11.2	93	75
Q4	1,324	19.6	159	169
P1	2,167	32.0	143	133
NOT(P1)	4,599	68.0	867	485
