# Supplementary material for: Qualitative and quantitative analysis of the proautophagic activity of Citrus flavonoids from Bergamot Polyphenol Fraction
Source: Data Brief. 2018 May 31;19:1327–34. doi: 10.1016/j.dib.2018.05.139 (PMC6140830; doi:10.1016/j.dib.2018.05.139)
Supplement: Supplementary file 16 — Supplementary material [file mmc16.pdf]

FACSDiva Version 6.1.2

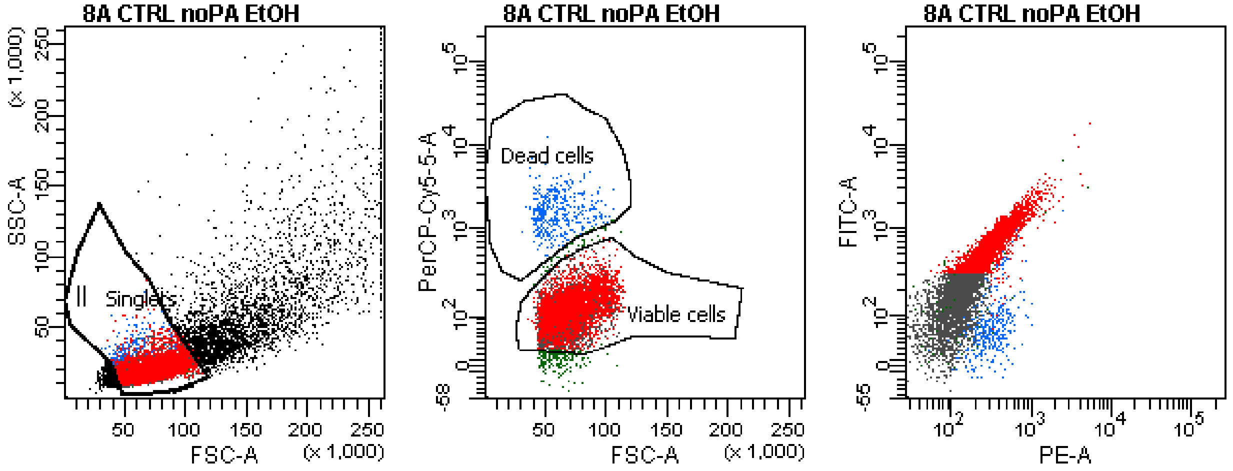

Tube: 8A CTRL noPA EtOH

Population	#Events	%Parent	%Total
All Events	10,000	####	100.0
Singlets	6,379	63.8	63.8
Dead cells	416	6.5	4.2
Viable cells	5,673	88.9	56.7
Q1	43	0.8	0.4
Q2	3,175	56.0	31.8
Q3	1,127	19.9	11.3
Q4	1,328	23.4	13.3
P1	2,565	45.2	25.7
NOT(P1)	3,108	54.8	31.1

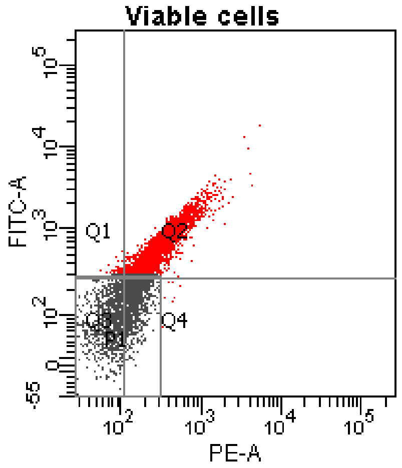

Tube Name: 8A CTRL noPA EtOH

Population	#Events	%Parent	FITC-A Mean	PE-A Mean
Singlets	6,379	63.8	419	261
Dead cells	416	6.5	147	357
Viable cells	5,673	88.9	446	258
Q1	43	0.8	315	92
Q2	3,175	56.0	687	368
Q3	1,127	19.9	98	72
Q4	1,328	23.4	170	157
P1	2,565	45.2	144	119
NOT(P1)	3,108	54.8	695	373
