# Supplementary material for: Qualitative and quantitative analysis of the proautophagic activity of Citrus flavonoids from Bergamot Polyphenol Fraction
Source: Data Brief. 2018 May 31;19:1327–34. doi: 10.1016/j.dib.2018.05.139 (PMC6140830; doi:10.1016/j.dib.2018.05.139)
Supplement: Supplementary file 17 — Supplementary material [file mmc17.pdf]

FACSDiva Version 6.1.2

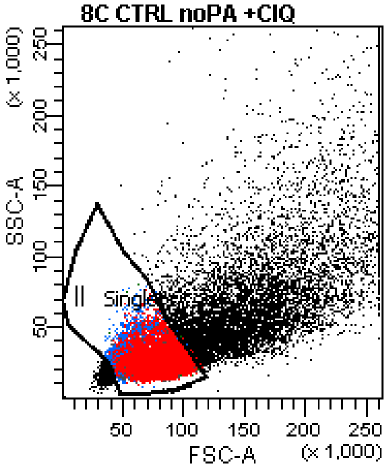

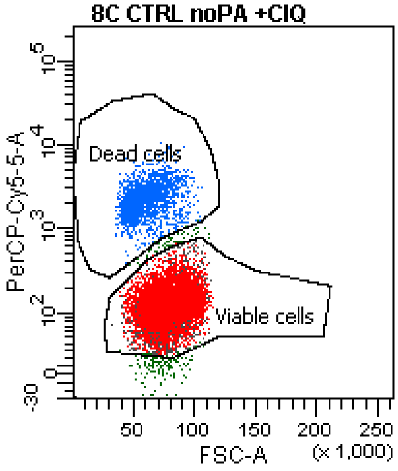

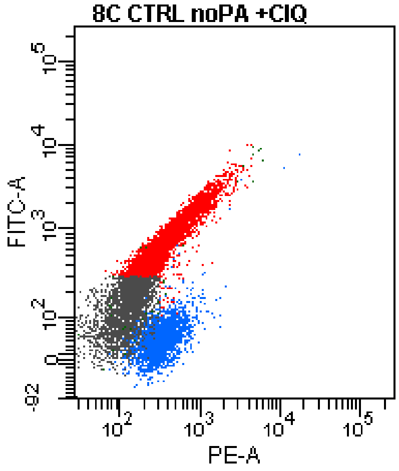

Tube: 8C CTRL noPA +CIQ

Population	#Events	%Parent	%Total
All Events	20,000	####	100.0
Singlets	11,209	56.0	56.0
Dead cells	2,503	22.3	12.5
Viable cells	8,473	75.6	42.4
Q1	25	0.3	0.1
Q2	5,896	69.6	29.5
Q3	888	10.5	4.4
Q4	1,664	19.6	8.3
P1	2,653	31.3	13.3
NOT(P1)	5,820	68.7	29.1

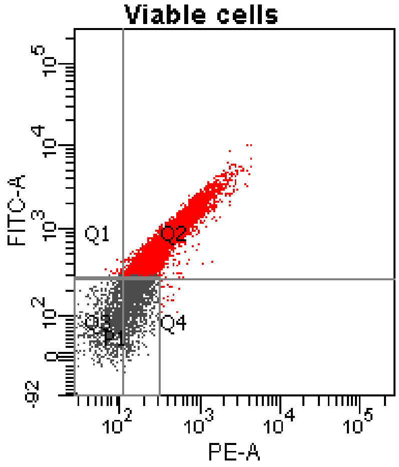

Tube Name: 8C CTRL noPA +CIQ

Population	#Events	%Parent	FITC-A Mean	PE-A Mean
Singlets	11,209	56.0	518	376
Dead cells	2,503	22.3	55	359
Viable cells	8,473	75.6	655	381
Q1	25	0.3	308	94
Q2	5,896	69.6	881	490
Q3	888	10.5	87	74
Q4	1,664	19.6	164	164
P1	2,653	31.3	143	132
NOT(P1)	5,820	68.7	888	495
